# (2*S*, 4*R*)-4-[^18^F]Fluoroglutamine for *In vivo* PET Imaging of Glioma Xenografts in Mice: an Evaluation of Multiple Pharmacokinetic Models

**DOI:** 10.1007/s11307-020-01472-1

**Published:** 2020-01-28

**Authors:** Maxwell WG Miner, Heidi Liljenbäck, Jenni Virta, Joni Merisaari, Vesa Oikonen, Jukka Westermarck, Xiang-Guo Li, Anne Roivainen

**Affiliations:** 1 Turku PET Centre, University of Turku, Kiinamyllynkatu 4-8, FI-20520 Turku, Finland; 2 Turku Center for Disease Modeling, University of Turku, FI-20014 Turku, Finland; 3 Turku Centre for Biotechnology, University of Turku and Åbo Akademi University, FI-20520 Turku, Finland; 4 Turku PET Centre, Åbo Akademi University, FI-20520 Turku, Finland; 5 Turku PET Centre, Turku University Hospital, FI-20520 Turku, Finland

**Keywords:** Fluoroglutamine, Glioma, Mice, Metabolism, Pharmacokinetic modeling

## Abstract

**Purpose:**

The glutamine analogue (2*S*, 4*R*)-4-[^18^F]fluoroglutamine ([^18^F]FGln) was investigated to further characterize its pharmacokinetics and acquire *in vivo* positron emission tomography (PET) images of separate orthotopic and subcutaneous glioma xenografts in mice.

**Procedures:**

[^18^F]FGln was synthesized at a high radiochemical purity as analyzed by high-performance liquid chromatography. An orthotopic model was created by injecting luciferase-expressing patient-derived BT3 glioma cells into the right hemisphere of BALB/cOlaHsd-Foxn1^nu^ mouse brains (tumor growth monitored *via in vivo* bioluminescence), the subcutaneous model by injecting rat BT4C glioma cells into the flank and neck regions of Foxn1^nu/nu^ mice. Dynamic PET images were acquired after injecting 10–12 MBq of the tracer into mouse tail veins. Animals were sacrificed 63 min after tracer injection, and *ex vivo* biodistributions were measured. Tumors and whole brains (with tumors) were cryosectioned, autoradiographed, and stained with hematoxylin-eosin. All images were analyzed with CARIMAS software. Blood sampling of 6 Foxn1^nu/nu^ and 6 C57BL/6J mice was performed after 9–14 MBq of tracer was injected at time points between 5 and 60 min then assayed for erythrocyte uptake, plasma protein binding, and plasma parent-fraction of radioactivity to correct PET image-derived whole-blood radioactivity and apply the data to multiple pharmacokinetic models.

**Results:**

Orthotopic human glioma xenografts displayed PET image tumor-to-healthy brain region ratio of 3.6 and 4.8 while subcutaneously xenografted BT4C gliomas displayed (*n* = 12) a tumor-to-muscle (flank) ratio of 1.9 ± 0.7 (range 1.3–3.4). Using PET image-derived blood radioactivity corrected by population-based stability analyses, tumor uptake pharmacokinetics fit Logan and Yokoi modeling for reversible uptake.

**Conclusions:**

The results reinforce that [^18^F]FGln has preferential uptake in glioma tissue *versus* that of corresponding healthy tissue and fits well with reversible uptake models.

**Electronic supplementary material:**

The online version of this article (10.1007/s11307-020-01472-1) contains supplementary material, which is available to authorized users.

## Introduction

Although relatively rare, malignant glioma development in humans often leads to a swift terminal outcome due to the aggressive nature of a large portion of the brain tumors [1, 2]. The complications accompanying intracranial surgery and the high instances of tumor relapse [3] reveal the importance of developing and rigorously testing improved imaging strategies. Detecting, characterizing, and monitoring these lesions can guide treatment strategies and ultimately lead to better patient outcomes. The compound (2*S*, 4*R*)-4-[^18^F]fluoroglutamine ([^18^F]FGln) has been shown to be particularly suitable for positron emission tomography (PET) imaging intracranial gliomas with the potential to differentiate between actively growing and clinically stable lesions [4]. Testing for its ability to image other types of cancers is ongoing [5].

**Fig. 1.**
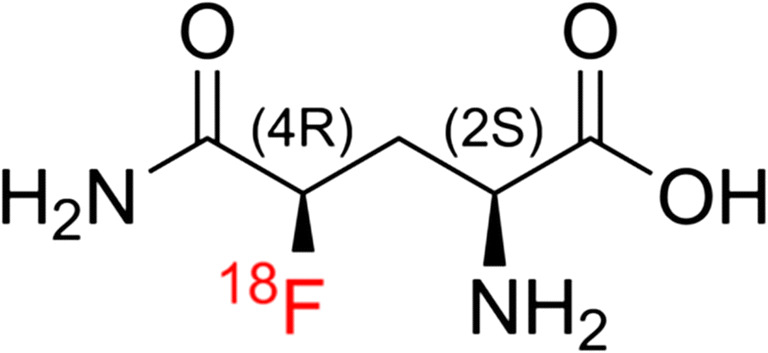
(2*S*, 4*R*)-4-[^18^F]fluoroglutamine chemical structure.

Upregulation of glutamine consumption has been long reported in various cancer cell lines occasionally earning them the classification of being “glutamine-addicted” [6–8]. As a substrate for the citric acid cycle, energetic metabolite, and protein building block, it is no surprise that there is a suggested relationship between glutamine uptake and current cellular metabolic levels [4]. Cellular import and export of glutamine occurs *via* many different transport proteins, though the most ubiquitous are classified as generalists and possess the ability to import a variety of similar but different amino acids [9, 10]. The F-18 fluorinated glutamine analogue, [^18^F]FGln (Fig. 1), has been shown to have comparable uptake kinetics of the natural version when contrasted to [^11^C]glutamine [11] which has made it a suitable imaging candidate in biological systems.

Radiolabelled energetic metabolite analogues, most notably: 2-deoxy-2-[^18^F]fluoro-d-glucose ([^18^F]FDG), have played a predominant role in PET imaging since 1976 [12]. Due to their intrinsic relation to cellular metabolic activity, they can accumulate faster in areas which require more energy. For organs with high basal levels of [^18^F]FDG uptake, such as the brain [13], detecting subtle increases in tracer accumulation can be an arduous task. Since the brain runs primarily on glucose-derived energy [13] and is usually relatively stable in terms of active cellular division [14] (during adulthood), it does not normally require large amounts of amino acids. In turn, an actively dividing glioma will require metabolites for both energy and amino acids for protein synthesis, revealing a possible high-contrast gradient ideal for PET imaging. Radiolabelled amino acids and analogues have proven to be part of an effective brain PET imaging strategy due to their relatively low basal level of uptake and ability to divulge insight into metabolic activity levels [15–17]. [^11^C]Methionine ([^11^C]Met) has been widely used in the European Union for acquiring human PET brain scans [18, 19] though, as with all C-11 tracers, presents some logistical limitations with the short 20-min half-life of the radionuclide.

[^18^F]FGln benefits from its analogue being both an energetic substrate [20] and protein building block. Combined with having low brain basal uptake [4, 21], the tracer has many positive qualities for imaging cancerous lesions in the brain. Early testing trials in humans suggest that PET images acquired with this tracer may allow oncologists to differentiate between actively growing and clinically stable lesions [4]. This is particularly important for treatment planning since aggressive treatment strategies need not be unnecessarily pursued, improving the quality of life for the patient. As clinical trials of [^18^F]FGln (spearheaded by Mark Dunphy, D. O., et al. in the USA, National Library of Medicine ID: NCT01697930) progress forward, continual animal studies allow for additional insight into the tracer’s limitations and the discovery of new imaging niches which may be filled (published investigated animal and tumor models: see Suppl. Table 1 in Electronic Supplementary Material (ESM), [4, 22–24]). Although not necessarily the final solution due to the ubiquity of which the native analogue (glutamine) is used throughout the mammalian body, [^18^F]FGln may represent the next step forward in PET imaging brain cancers due to the particularly low brain basal uptake level providing exceptional tumor tissue contrast [4]. Further variants in structure: alkyl chains [25], poly peptide additions [26], and a boron trifluoride variant [27] are continuously being investigated in search of improvements in imaging and *in vivo* stability.

Herein, we report the radiosynthesis of [^18^F]FGln in a custom-made radiosynthesis device [28] and PET imaging of two separate glioma models in mice. We also describe four pharmacokinetic model evaluations (with and without bioavailable and metabolite corrections to the blood activity inputs) with the thought that future changes to the tracer structure will have a rich source of uptake parameters to quantify *in vivo* flux changes.

## Methods

### Animal Models

The intracranial glioma model was made by injecting female BALB/cOlaHsd-Foxn1^nu^ (athymic nude, Institute of Animal Genetics, Edinburgh, UK) mice with patient-derived BT3 glioma cells [29] labeled with luciferase into the frontal right hemisphere of the brain at 9 weeks old. Tumors were monitored for growth *via in vivo* bioluminescence detection and subjects PET imaged after 12 days.

The subcutaneous tumor model was created by injecting rat BT4C glioma cells subcutaneously into the hind leg and neck region of six athymic nude (Foxn1^nu/nu^, Envigo, Gannant, France) mice at 7 weeks old. Tumor growth was visually tracked and subjects PET imaged after 5 weeks. See Electronic Supplemental Materials (ESM) for detailed animal models.

Tracer *in vivo* stability studies were performed with 12 mice consisting of two groups; 6 1-year-old male C57BL/6J and 6 3-month-old female Foxn1^nu/nu^ mice.

The animals were sacrificed after imaging (or the final metabolism blood collection point) under deep isoflurane anesthesia (4 %) *via* cervical dislocation after the preceding cardiac puncture to extract blood samples. All animal work was approved by the National Animal Experiment Board of Finland and the Regional State Administrative Agency for Southern Finland (permission no. ESAVI/12691/04.10.07/2017 and 4161/04.10.07/2015) while being carried out in compliance with the EU directive relating to the conduct of animal experimentation.

### (2S, 4R)-4-[^18^F]Fluoroglutamine Preparation

The radiosynthesis of (2*S*, 4*R*)-4-[^18^F]fluoroglutamine was accomplished with similar methods to previous described syntheses [11] with some changes such as not using a vacuum pump and increasing drying temperatures. See Suppl. Methods in ESM for a detailed list of modifications, quality control methods, and representative chromatograms (Suppl. Figs. 1 and 2 in ESM).

### *In Vivo* Imaging and Analysis

One to two mice were imaged at a time while under 2 % isoflurane anesthesia with an Inveon multimodality small animal PET/CT scanner (Siemens Medical Solutions, Munich, Germany) commencing with an attenuation CT scan to correct the PET scan data. Ten to twelve megabequerels of the tracer was injected into the tail vein, and the subject(s) were imaged over a 60-min period in list mode dividing framing into 6 × 10 s, 4 × 60 s, 5 × 300 s, 3 × 600 s. Obtained sinograms were reconstructed using the ordered-subset expectation maximization 2-dimensional algorithm to obtain the final PET images and back-corrected for radionuclide decay.

Image analysis was performed using CARIMAS software published and freely distributed by the Turku PET Center (http://turkupetcentre.fi/carimas/). CT scans were used as rough anatomical reference while tumor regions of interest (ROI) were hand-drawn based upon observed spikes of radioactivity in the PET images at locations correlating with recorded tumor grafts. Control muscle ROIs were drawn on the side opposite to the tumor in the leg flank muscle (based on CT) while the blood ROI was drawn based upon heart anatomical location and observed PET image radioactivity spike during the initial 20 s of imaging in the left ventricle. Standardized uptake values (SUV) were calculated with respect to ROI volume, mean and max tumor radioactivity, total injected radioactivity dose (corrected for tail, cannula, and syringe residual radioactivity), and animal weight, and were additionally presented as tumor-to-background ratio (TBR, tumor-to-healthy brain region for intracranial gliomas and tumor-to-muscle for subcutaneous gliomas) as defined by recent glioma research literature standards [30]. Mean and maximum TBR values were reported as ROI radioactivity per volume divided by mean background radioactivity per volume. The blood radioactivity SUV and gender-specific metabolite-corrected blood radioactivity SUV were then applied to multiple pharmacokinetic models to characterize the flux of the tracer (further described in the “Modeling” section).

### *In Vivo* Stability Analysis

Twelve non-tumor-bearing control mice (6 C57BL/6J and 6 Foxn1^nu/nu^) were injected with 9–14 MBq of tracer before a 200-μL blood sample was drawn into a heparinized vial and kept on ice. After 15 min, a second sample was collected during animal sacrifice. Whole blood was weighed and measured for radioactivity before being centrifuged at 730×*g* and 5 °C for 5 min, separated into plasma and blood cell fractions, and then separately weighed and measured. The values were used along with age, gender, and animal model-specific hematocrit values (reported by suppliers) to calculate the plasma radioactivity fraction of the whole-blood radioactivity (Eq. 1) [31]:

1 $$ \mathrm{Plasma}\ \mathrm{Activity}\ \mathrm{Fraction}=\frac{C_p}{C_{\mathrm{RBC}}+{C}_p}=\frac{\rho_p{C}_p\left(\mathrm{HCT}\right)}{\rho_b{C}_b-{\rho}_p{C}_p\left(1-\mathrm{HCT}\right)+{\rho}_p{C}_p\left(\mathrm{HCT}\right)} $$

where *C* = radioactivity concentration, *ρ* = density, and HCT = hematocrit. Subscripts denote the following: *p* = plasma, RBC = red blood cell, and *b* = whole blood.

Aliquots of plasma were precipitated with 2.5 × (volume) methanol, vortexed, and centrifuged at 11,000×*g* for 5 min. The pellet and supernatant fractions were separated and measured for radioactivity to give the radioactivity fraction of the plasma that was trapped or bound to proteins (Eq. 2):

2 $$ \mathrm{Unbound}\ \mathrm{plasma}\ \mathrm{fraction}=\frac{C_{\mathrm{sup}}}{C_{\mathrm{pel}+{C}_{\mathrm{sup}}}} $$

where *C* = radioactivity of subscripts which denote the following: sup = supernatant and pel = pellet.

Aliquots of the precipitated plasma supernatant were assayed for parent-tracer stability *via* previously established high-performance liquid chromatography (HPLC) methods [24] (Suppl. Methods in ESM). The results from the three assays, which constitute fractions of tracer availability, were individually factored together for each time point to give an estimate of the total bioavailable [^18^F]FGln fraction of whole-blood radioactivity. The curves were approximated as result Eqs. 3 and 4, and the uncorrected and corrected blood radioactivities were then both used as separate inputs for all of the models.

### *Ex Vivo* Studies

See Suppl. Materials in ESM for detailed descriptions of biodistribution, stability, cryosectioning, autoradiography, and histology.

### Statistical Methods and Modeling

Using the heart’s left ventricle ROI from the PET data as an uncorrected radioactivity level input and the bioavailable parent-tracer fraction (results: Eqs. 3 and 4 applied to the uncorrected input) as the corrected input, multiple models were investigated to characterize the tissue uptake dynamics. Two-compartment modeling and Logan, Patlak, and Yokoi plots were used to separately model the pharmacokinetics for the gliomas (both subcutaneous and orthotopic loci) and their respective control tissues (healthy muscle and healthy brain, respectively). Where indicated, means plus/minus standard deviations are reported. Difference in parameters for uncorrected *versus* correct and target tissue *versus* their respective control regions were tested with students’ paired *t* tests with *P* values lower than 0.05 being considered significant.

## Results

### Tracer Quality Control and Shelf Life Analyses

On average (*n* = 3), the final product was produced with a radioactivity of 446 ± 49 MBq (decay-corrected to 952 ± 105 MBq) starting from 12.5 GBq of [^18^F]F^−^, giving a decay-corrected radiochemical yield of 7.1 ± 1.4 % starting from end-of-bombardment. The average (*n* = 3) enantiomeric purity produced 97.2 ± 0.6 % of the desired 2*S*, 4*R* compound with the rest being the 2*S*, 4*S* enantiomer. The overall average (*n* = 3) radiochemical composition of the product was 93.5 ± 0.6 % (2*S*, 4*R*)-4-[^18^F]fluoroglutamine, 2.7 ± 1.1 % (2*S*, 4*S*)-4-[^18^F]fluoroglutamine, and 3.9 ± 0.9 % other (Suppl. Fig. 1 in ESM).

### *In Vivo* Stability Analysis

Plasma radioactivity fraction of the whole blood (Suppl. Fig. 3a in ESM) showed a decrease over time which equated inversely to erythrocyte uptake. The plasma precipitation assay results (Suppl. Fig. 3b in ESM) showed that after 60 min, roughly 25 % of the radioactivity in the plasma was either bound to or incorporated into proteins. Of the unbound plasma radioactivity, the HPLC analysis of this fraction showed a difference in stability between groups (Suppl. Fig. 3c in ESM).

**Fig. 2.**
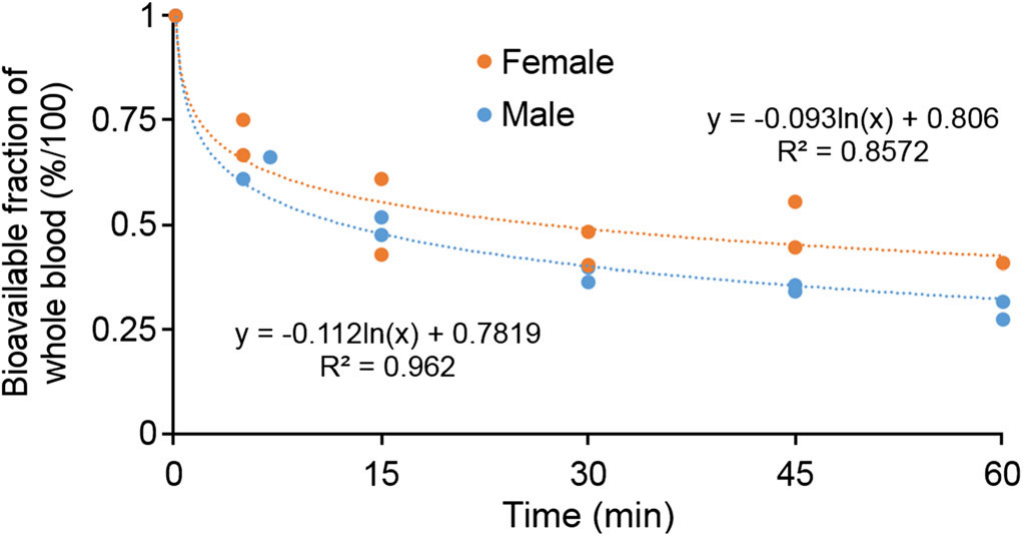
Parent-tracer bioavailable fraction of whole blood (calculated by multiplying the Suppl. Fig. 3a–c in ESM sample-specific data points) for 1-year-old male C57BL/6J and 3-month-old female Foxn1^nu/nu^ mice.

Points of the available fractions (Suppl. Fig. 3a–c in ESM) for each sample were factored together to produce overall estimates of the bioavailable [^18^F]FGln fraction of whole blood (Fig. 2). The resulting Eqs. 3 and 4 were fitted to approximate this fraction at each time point (in minutes) to apply to modeling input data.

3 $$ \mathrm{Male}\ \mathrm{Bioavailable}\ \mathrm{Fraction}\ (t)=\left(\mathrm{Tracer}\ \mathrm{Purity}\right)\left(-0.112\right)\ln (t)+0.7819\kern0.5em {R}^2=0.962 $$

4 $$ \mathrm{Female}\ \mathrm{Bioavailable}\ \mathrm{Fraction}\ (t)=\left(\mathrm{Tracer}\ \mathrm{Purity}\right)\left(-0.093\right)\ln (t)+0.806{R}^2=0.8572 $$

Blood (heart left ventricle, both uncorrected and bioavailability-corrected), bone, glioma, and control regions in dynamic PET image data were fitted with multiple models to produce uptake pharmacokinetic values which agree with the current understanding of glutamine transport and metabolism. Logan plots for a reversible uptake model fit well and displayed significant (*p* < 0.002) increases in distribution volume of the tumors *versus* the control muscle. Patlak plots (Suppl. Fig. 4 in ESM) showed no significant differences in irreversible uptake kinetics for the tumors *versus* the control areas, providing little additional insight into the metabolic changes. When corrected for bioavailable parent-tracer fraction, the Ki values overlapped further changing the *T* test *p* values from roughly 0.2 to 0.9. The large increase in irreversible bone uptake flux supported the hypothesis that the defluorinated [^18^F]fluoride was binding irreversibly to the bone which, in combination with the biodistribution, supported that defluorination is occurring *in vivo*.

### Orthotopic Glioma Images and Analyses

**Fig. 3.**
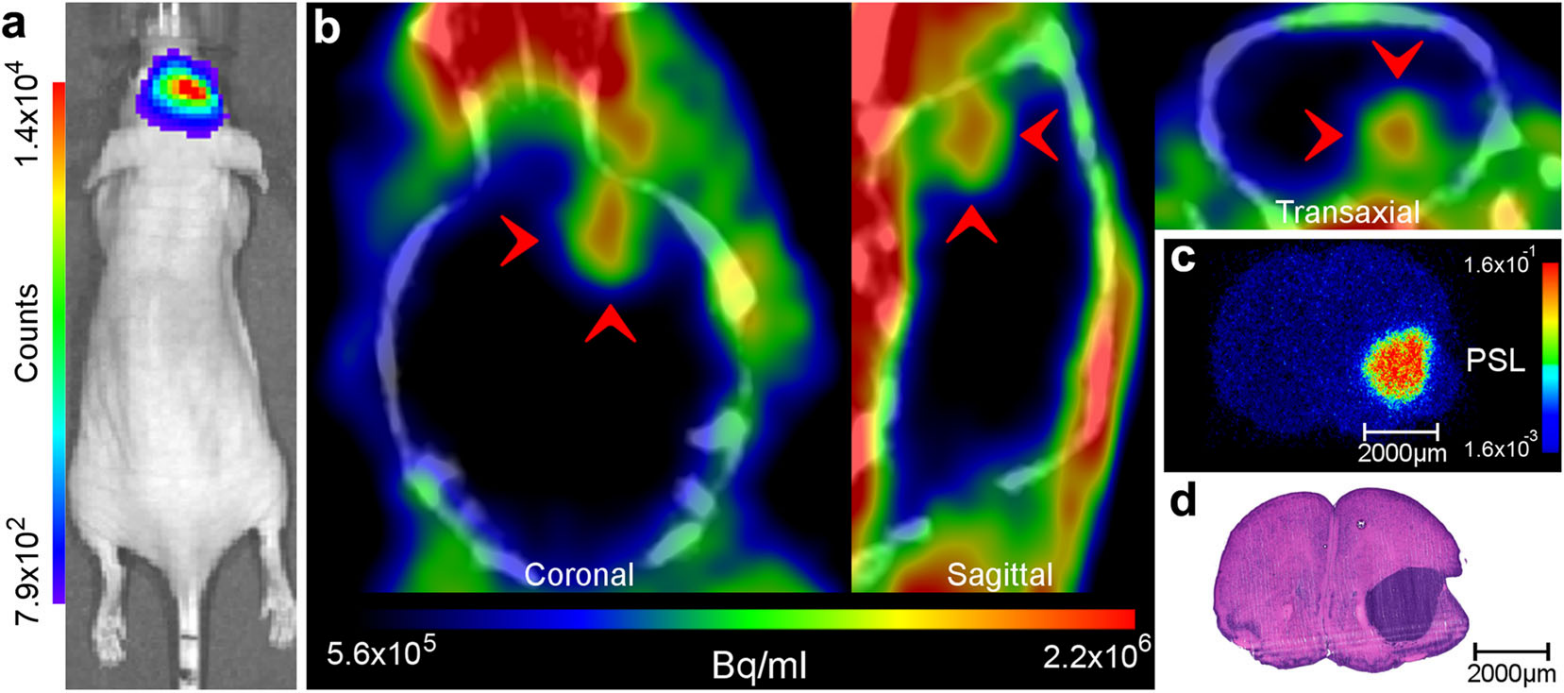
Multimodal image array of a BALB/cOlaHsd-Foxn1^nu^ mouse with a patient-derived BT3 glioma labeled with luciferase orthotopically grafted into the right hemisphere of the brain. **a***In vivo* bioluminescence. **b***In vivo* [^18^F]FGln-injected PET image slices (sum of frames from 5 to 25 min) with tumor indicated by red arrows. **c***Ex vivo* autoradiograph of a (transaxial) cryosection. **d** Light micrograph (hematoxylin-eosin stain) of the cryosection.

**Fig. 4.**
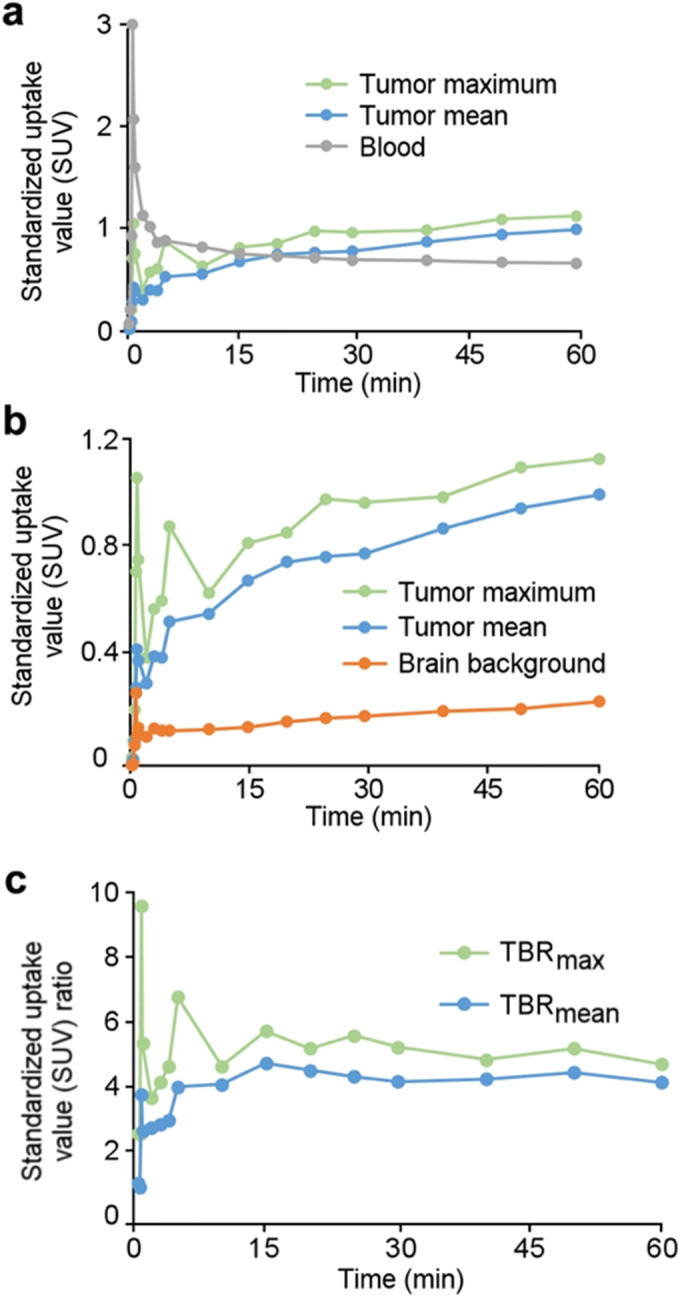
Array of PET time-radioactivity **a**, **b** curves, **c** ratio curves, for a [^18^F]FGln-injected BALB/cOlaHsd-Foxn1^nu^ mouse (PET images depicted in Fig. 3). TBR_mean_ = mean tumor radioactivity to mean brain background radioactivity ratio and TBR_max_ = maximum voxel radioactivity to mean brain background radioactivity ratio.

The orthotopic xenografts of luciferase-expressing patient-derived BT3 glioblastoma cells [29] grown in BALB/cOlaHsd-Foxn1^nu^ mouse brains produced strong *in vivo* bioluminescence detections and displayed clear differentiation of the tumor mass in *in vivo* PET and *ex vivo* autoradiography images (Fig. 3 and Suppl. Fig. 5 in ESM). Time-radioactivity curves (TACs) of tumor, healthy brain tissue, and heart left ventricle (blood) presented in Fig. 4 (Suppl. Fig. 6 in ESM) suggested that the PET image TBRs peaked between 15 and 40 min. *Ex vivo* light micrographs confirmed (Fig. 3d) the presence of the gliomas while autoradiographs of the cryosections displayed an average (*n* = 3 subjects, 36 sections), TBR of 8.9 ± 1.1 (range 7.2–11.0) (sample: Fig. 3c and Suppl. Fig. 4c in ESM). A Summary of the model data is included in Table 1.

**Table 1.** Orthotopic glioma data summary

Subject	Bioluminescence (p/s)	PET TBR_mean_	Autoradiography TBR_mean_^a^
A	1.6 × 10^7^	4.8	8.9 ± 1.1 (range 7.2–10.5)
B	9.7 × 10^6^	3.6	9.9 ± 0.7 (range 8.9–11.0)
C	1.6 × 10^5^	N/A^b^	7.8 ± 0.4 (range 7.3–8.4)

^a^*n* = 12 brain sections per subject, ^b^image acquisition complications

### Subcutaneous Glioma Images and Analyses

**Fig. 5.**
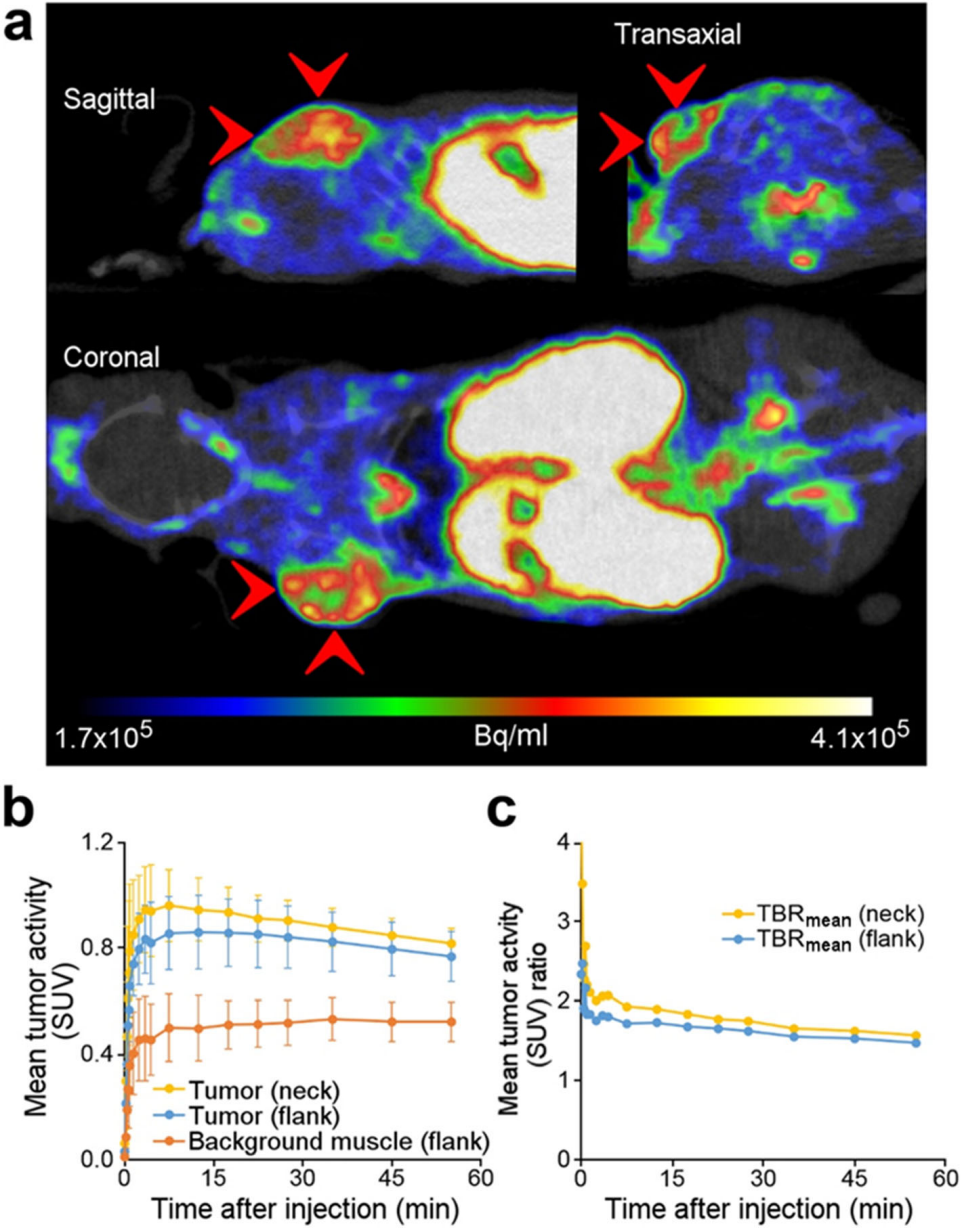
**a** PET image slices from a whole body [^18^F]FGln PET/CT image of a Foxn1^nu/nu^ mouse (time-weighted mean of frames from 1 to 40 min post-injection) with a subcutaneously (neck) xenografted BT4C glioma pointed out with red arrows. **b** Average (*n* = 6) [^18^F]FGln time-radioactivity curves of the subcutaneous model. **c** Average (*n* = 6) mean tumor to background ratios (TBR_mean_).

TACs (Fig. 5b) extracted from the PET images show that the average (*n* = 6) uptake in both the neck and flank BT4C tumor xenografts was significantly (*p* < 0.02) higher than that in the healthy flank muscle. When tracer uptake plateaued at around 12.5 min, the tumor-to-background muscle ratios were 1.8 ± 0.6 (range 1.3–3.0) and 2.0 ± 0.8 (range 1.4–3.4) for neck and flank loci, respectively (Fig. 4c). See Suppl. Fig. 7 in ESM for autoradiography and histology example.

### Modeling

**Fig. 6.**
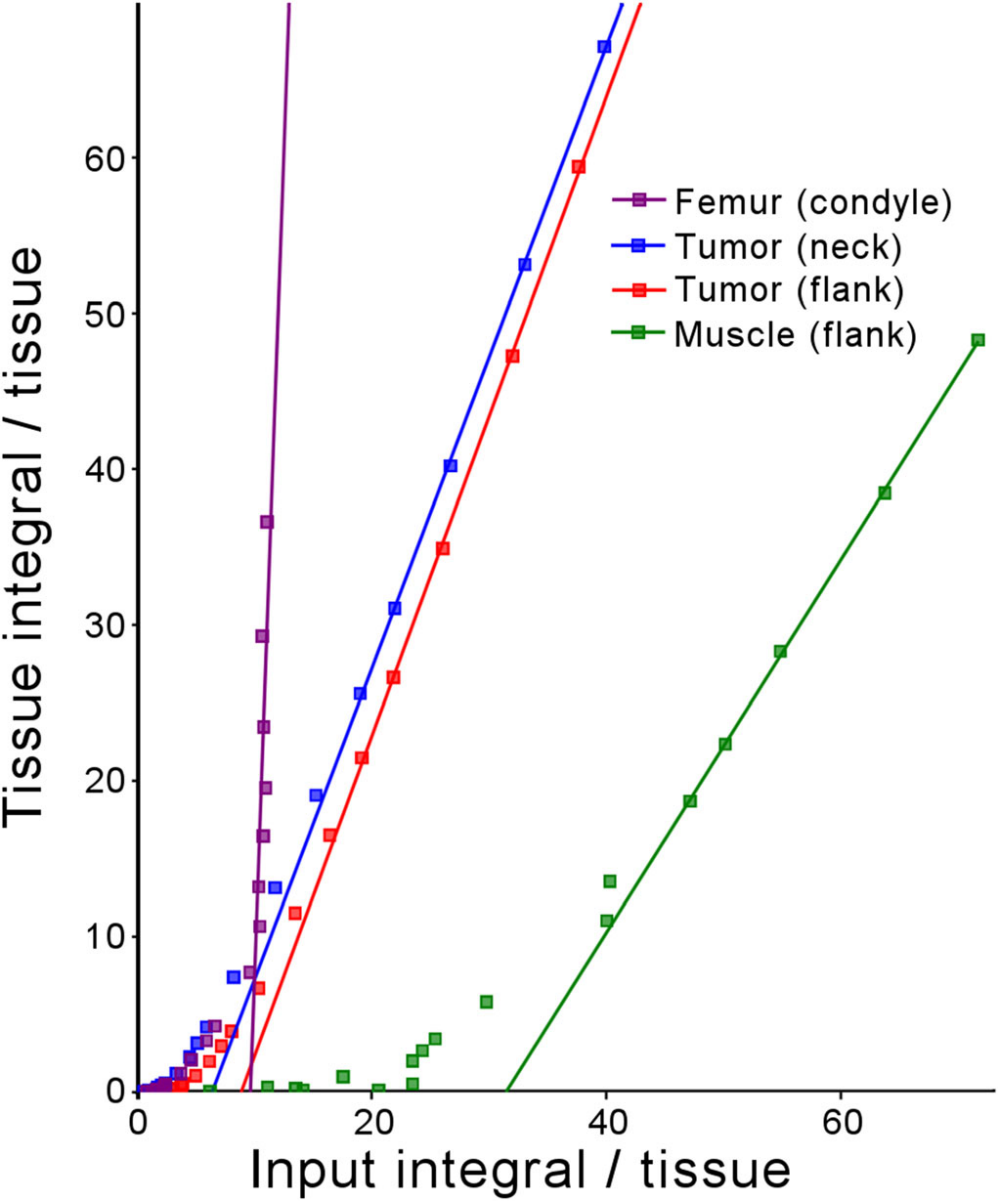
Sample Logan plot of dynamic PET image tissue uptake with population-based metabolite-corrected bioavailable fraction of arterial blood as the input.

Logan plots (sample: Fig. 6) showed that there was a unanimous increase in tracer uptake net flux (slope) in tumors *versus* the control tissues. Average distribution volume from the plots (Table 2, both metabolite-corrected and uncorrected inputs) showed a significant (*p* < 0.003) increase in tumor uptake rate *versus* control muscle. Differences between the metabolite-corrected and uncorrected flux and uptake parameters of the tumor tissue were also significant (*p* < 0.00004). Of the multiple pharmacokinetic models investigated, the Logan, Yokoi, and 2-compartment modeling suggested that reversible kinetics play a more dominant role (2 orders of magnitude higher) *versus* an irreversible (Patlak) uptake model in all investigated (excluding osseous) tissues.

**Table 2.** Logan plot (reversible uptake model) result summary

Region of interest (*n* = 6)	AB distribution volume (ml)	PC distribution volume (ml)
Flank tumor	1.13 ± 0.09	1.96 ± 0.15
Flank muscle	0.77 ± 0.13	1.38 ± 0.23
Neck tumor	1.21 ± 0.08	2.08 ± 0.13
Bone (femoral condyle)	5.11 ± 2.19	13.47 ± 7.70

*AB*, arterial blood radioactivity used as input; *PC*, plasma-corrected (population-based metabolite-corrected fraction of the arterial blood radioactivity) used as input

Paired student’s *t* tests comparing distribution volume of tumors *versus* flank muscle (both AB and PC) and comparing AB inputs *versus* PC inputs were all significant (*p* < 0.003 and *p* < 0.00004, respectively)

See Suppl. Figs. 8–10 and Suppl. Tables 2–6 in ESM for complete modeling data (Patlak, Yokoi, and 2-compartment modeling).

### Biodistribution

*Ex vivo* biodistribution studies confirmed higher subcutaneous tumor uptake than control muscle tissue with high uptake in the pancreas (6-fold higher than tumor) and high uptake in the small intestine, skull (bone), and femur (bone + marrow). See Suppl. Fig. 11 in ESM for detailed *ex vivo* biodistribution results.

## Discussion

Survival rates and treatment strategies for glioma-bearing patients are heavily tied to the grading and resulting aggressiveness of the brain tumors. Using non-invasive PET imaging to divulge biochemical characteristics of the tumors can offer critical insight which can impact treatment planning. *In vivo* evaluation of the uptake kinetics, biodistribution, metabolism, and PET imaging capabilities of [^18^F]FGln provides validation of reported efficacy while investigating its limitations.

The synthesis of the tracer was accomplished in close accordance to literature methods though with different instrumentation and modified technical steps, *i.e.*, without the use of a vacuum pump by increasing the duration and temperature of the drying phases under nitrogen gas flow. The [^18^F]FGln tracer was then successfully evaluated *in vivo* within two previously uncharacterized tumor models showing favorable PET imaging characteristics with particularly suitable imaging properties for orthotopic gliomas.

The subcutaneous model produced results which were not quite as promising as the intracranial tumors and was hypothesized to be related to the much higher basal uptake levels of muscle tissue (Biodistribution in Suppl. Fig. 11 in ESM). It was still possible, however, to discern the glioma from the surrounding tissue (Fig. 5) though tracer uptake was non-homogenous in some gliomas causing a drop in reported average TBR values. The shell-like distribution observed in PET images of some BT4C tumors (confirmed *via ex vivo* autoradiography) was at first thought to be indicative of central mass necrosis though *ex vivo* studies did not support this. When examining light micrograph scans, no obvious localized signs of cell death were present in the cryosections which exhibited low uptake in comparison with the high uptake regions of the tumors (example slide: Suppl. Fig. 7 in ESM).

Although the HPLC analysis of the plasma supernatants divulges the parent-tracer purity at the listed time points, the fleeting nature of the free [^18^F]fluoride, *e.g.*, fast bone uptake, makes it difficult to obtain a complete model of the tracer kinetics. Furthermore, the first metabolite ([^18^F]FGlu) [22] is also taken up by cells with its own rate [32], which adds some obscurity to the true values. Application of the bioavailable fraction equation to the whole-blood radioactivity PET image curves for modeling purposes was matched by gender instead of mouse strain which, at first-glance, denotes a major limitation. Significant gender differences have been reported in highly relevant metabolic rates and enzyme expression in the liver which is the organ primarily responsible for amino acid processing [33, 34] which we considered to justify the grouping. Further, the main gender metabolism difference is visible (Suppl. Fig. 3c in ESM) in the plasma-free fraction where a faster decrease in male parent-tracer levels would be present from a higher metabolic rate.

Modeling of the tracer’s pharmacokinetics was assessed with four separate models, each with both the arterial blood and corrected fraction of the said blood as the input. In most cases, all models (reversible and irreversible) fit well with *r*-values primarily close to 1 and Akaike criterion of − 100 to − 120 for 2-compartment modeling with some exceptions. Patlak (irreversible uptake) modeling fit poorly for soft tissues until corrected inputs were used, and interestingly, the rate of irreversible uptake was found to be similar for tumor and muscle areas. The irreversible uptake rate, however, was calculated to be roughly 15 times lower than the reversible uptake rates reinforcing the notion that the tracer uptake is primarily reversible. The reversible uptake models were largely in agreeance for flux rates, and therefore, all data tables have been reported (Suppl. Tables 2–6 in ESM) with the thought that future preclinical studies of similar tracers in mouse models could directly compare their pharmacokinetics. Bone (femoral condyle) tissue was found to behave in reverse to that of the soft tissues investigated and fit better with an irreversible uptake model which divulged a two order of magnitude increase in uptake *versus* calculated reversible uptake values. This supported the lack of *in vivo* stability in the form of defluorination and agreed with current understanding of [^18^F]FGln metabolomics.

While the fact that metabolism is occurring does not come as a surprise considering that the compound is based upon a widely used natural metabolite, it is unfortunate that there have yet to be developments akin to [^18^F]FDG which render the compound biologically inert or difficult to be metabolized. The enzyme shown to be at least partially responsible for this defluorination (after it has been converted into (2*S*, 4*R*)-4-[^18^F]fluoroglutamate) is alanine aminotransferase which, in a stroke of unfortunate luck, processes and defluorinates the compound 5.6 times more efficiently than its non-fluorinated natural counterpart [32].

Although representing a small sample size, these initial results look very promising and produced uptake ratios well above the Society of Nuclear Medicine and Molecular Imaging thresholds [30] for other well-established tracers. Other currently used tracers for glioma imaging, namely [^18^F]FDG and [^11^C]Met, suffer differing limitations which [^18^F]FGln may address. Having orders of magnitude lower basal uptake in the brain than [^18^F]FDG and a longer half-life than [^11^C]Met reveal imaging niches for [^18^F]FGln to fill. Further research is being actively pursued to contrast its abilities in a direct comparison with these established routine-use clinical radiopharmaceuticals.

## Conclusions

The (2*S*, 4*R*)-4-[^18^F]fluoroglutamine compound was successfully prepared and tested for the first time at the Turku PET Center, demonstrating the ability to produce a high-purity compound with a custom-made radiosynthesis device. The tracer was safely and successfully utilized for *in vivo* studies in mice to PET image gliomas of different strains and grafted loci. *In vivo* stability analyses allowed for population-based metabolic corrections to approximate parent-tracer bioavailability in the whole blood which, when combined with PET image tumor uptake data, allowed for a model of the kinetics to be assessed and fit well with reversible uptake models (Logan, Patlak, and 2-compartment modeling).

Despite the discussed drawbacks due to tracer metabolism and defluorination, it is possible that [^18^F]FGln still has the potential to be a useful imaging compound especially with respect to brain tumors. The low basal level of brain uptake allows for the tumor-localized accumulation of tracer to be detected with high contrast. Further study is warranted and being actively pursued internationally as the tracer makes the delicate transition into clinical use. Regardless of human trial outcomes, its potential to be used for other non-cancer metabolism and amino acid import/export investigations will likely retain a niche for [^18^F]FGln to fill.

## Electronic Supplementary Material

ESM 1 (PDF 1.31 mb)
