# Supplementary material for: (2S, 4R)-4-[18F]Fluoroglutamine for In vivo PET Imaging of Glioma Xenografts in Mice: an Evaluation of Multiple Pharmacokinetic Models
Source: Mol Imaging Biol. 2020 Jan 28;22(4):969–78. doi: 10.1007/s11307-020-01472-1 (PMC7343746; doi:10.1007/s11307-020-01472-1)
Supplement: Supplementary file 1 — (PDF 1.31 mb) [file 11307_2020_1472_MOESM1_ESM.pdf]

Supplemental Material

(2*S*, 4*R*)-4-[^{18}F]Fluoroglutamine for *In Vivo* PET Imaging of Glioma Xenografts in Mice; An Evaluation of Multiple Pharmacokinetic Models

Maxwell WG Miner,[1] Heidi Liljenbäck,[1,2] Jenni Virta,[1] Joni Merisaari,[3] Vesa Oikonen,[1] Jukka Westermarck,[3] Xiang-Guo Li,[1,4] Anne Roivainen[1,2,5]

[1]*Turku PET Centre, University of Turku, FI-20520 Turku, Finland*

[2]*Turku Center for Disease Modeling, University of Turku, FI-20014 Turku, Finland*

[3]*Turku Centre for Biotechnology, University of Turku and Åbo Akademi University, FI-20520 Turku, Finland*

[4]*Turku PET Centre, Åbo Akademi University, FI-20520 Turku, Finland*

[5]*Turku PET Centre, Turku University Hospital, FI-20520 Turku, Finland*

Additional Methods

[^{18}F]FGln Synthesis Modifications

Following the steps outlined previously [11], the synthesis was accomplished with changes to the two key drying steps. No vacuum pumps were employed for either and instead, the first drying step of the eluted [^{18}F]fluoride from the quaternary methyl amine (QMA) anion exchange cartridge elution solution was done under continuous nitrogen flow (70 mL/min) at 118°C for roughly 29 minutes. It was found that the angles of the inflow and outflow needles impacted drying speed heavily and that positioning them to constructively create a vortex in the vial sped up the process. The trifluoroacetic acid (TFA) deprotection step towards the end of the synthesis was performed with half of the reported amounts of TFA + anisole solution (now 595 µL + 5 µL) under increasing nitrogen gas flow (60 mL/min for 3 minutes, then 110 mL/min for the remaining 2 mins) while heating to 60°C. The radiosynthesis was fitted into a custom-made device and the details of the device setup were similar as previously reported [28].

Quality Control and Shelf Life Analyses

Quality control analyses were performed immediately after synthesis with established methods published by Qu W. *et al* 2012 [11] with corresponding chromatograms and retention times used for corroborating peak identities. A Chirex Phenomenex 3126 (D)-Penicillamine 250 × 4.6 mm column running with an isocratic mobile phase of 1 mM Copper sulfate ($CuSO_4$) was used at a constant flow rate of 1 mL/min. Degradation analyses were performed by placing roughly 500 MBq in 2 mL phosphate-buffered saline at room temperature, pH 6.5 and 8 for 4 hours. A small aliquot was then extracted every hour for HPLC analysis via the same quality control method.

Animal Models

The intracranial glioma model was made with patient-derived BT-3 [29] glioma cells labeled with luciferase which were injected into 9 week old female BALB/cOlaHsd-Foxn1^{nu} mice (athymic nude, Institute of Animal Genetics, Edinburgh, UK). Under isoflurane anesthesia, a 0.5 mm incision was made to the skin covering the skull and a small hole (Ø 0.2 mm) was drilled above the injection site. Inoculation was performed slowly over 5 minutes and the needle remained 5 minutes after the injection (coordinates: bregma: 1 mm anterior, 2 mm to right, 3 mm depth from the skull). The wound was then sutured and the growth later monitored via *in vivo* bioluminescence detection (IVIS Spectrum system, Perkin Elmer, Hopkinton, MA, USA) before being PET imaged after 12 days.

The subcutaneous tumor model was created by suspending 250,000 rat BT4C glioma cells (originally extracted from BDIX rat strain) in 50 or 100 µL aliquots of 50% cell culture medium and 50% Matrigel. The suspension was injected subcutaneously into the hind leg and neck region of six athymic nude 7 week old mice (Foxn1$^{nu/nu}$, Envigo, Gannat, France) under isoflurane anesthesia. Tumor growth was visually tracked and the 6 mice (35.6 g ± 2.2 g) were imaged at approximately after 5 weeks when all of the tumors had grown to a size greater than 1 cm in diameter as measured externally with a caliper.

Ex Vivo Biodistribution

The listed organs; tumor(s), flank muscle, whole brain, skull (bone), femur (bone + marrow), white and brown adipose tissue, testis, epididymis, ovaries, uterus, heart, lungs, liver, spleen, pancreas, kidneys, stomach (empty), small intestine (empty), and large intestine (empty) were removed after animal sacrifice. They were then individually weighed and measured for radioactivity levels before being back corrected for decay time, amount of tracer injected, and the weight of the subject.

Ex Vivo Cryosectioning, Autoradiography, and Histology

Organs of interest were placed on ice immediately after dissection and biodistribution measurements. The samples were then frozen under liquid nitrogen and sliced with a Leica CM3050S cryotome into alternating 20 and 8 µm slices for autoradiography and potential future immunohistochemical investigations (respectively) before being placed on Menzel Superfrost Plus (Thermo Fisher) slides. 20 µm samples were exposed on a Fuji BAS-TR2025 imaging plate for 3-4 hours before developing the plates in a Fujifilm BAS-5000 system (Fujifilm, Tokyo Japan) to obtain autoradiographs. The slides were temporarily stored at -70 ^{o}C before being fixed and stained with hematoxylin-eosin before digital scanning with a Panoramic 250 Flash III slide scanner (3DHistech, Budapest, Hungary)

Additional Results and Discussion

Supplemental Table 1. Summarized information of previously published [^{18}F]FGln animal study models

Tumor cell line	Tumor origin	Animal model	Tumor locations	Reference
9L gliosarcoma	Rat	F344 rat	s.c.	[22]
M/tomND (spt.) mammary adenocarcinoma	Human	ICR mouse (spt.)	variable (spt.)	
TS603 (IDH1 R132H) glioma	Human	SCID mouse	intracranial, s.c.	[4]
RCAS-PDGF, (PTEN $^{-/-}$) glioblastoma	Human	SCID mouse	intracranial	
U87-MG (PTEN $^{-/-}$) glioma	Human	SCID mouse	s.c.	
TS543 (PDGFRA, PTEN $^{-/-}$) glioma	Human	SCID mouse	s.c.	
TS598 (EGFR) glioma	Human	SCID mouse	s.c.	
H520 squamous cell carcinoma	Human	Foxn1^{nu} mouse	s.c.	[23]
COLO-205 colon adenocarcinoma	Human	Foxn1^{nu} mouse	s.c.	
HCT-116 colon carcinoma	Human	Foxn1^{nu} mouse	s.c.	
HCC1806 breast ductal carcinoma	Human	Foxn1^{nu} mouse	s.c.	[24]
HCC38 breast ductal carcinoma	Human	Foxn1^{nu} mouse	s.c.	
MCF-7 mammary adenocarcinoma	Human	Foxn1^{nu} mouse	s.c.	

SCID = severe combined immunodeficient; s.c. = subcutaneous; spt. = spontaneous.

Quality Control and Shelf Life Analysis

At pH 6.5 the product showed no appreciable signs of degradation after 500 MBq was left at room temperature four hours. At pH 8 roughly 15% of the tracer had degraded after two hours under similar conditions emphasizing the necessity of proper pH balancing before batch release to imaging facilities (data not shown).

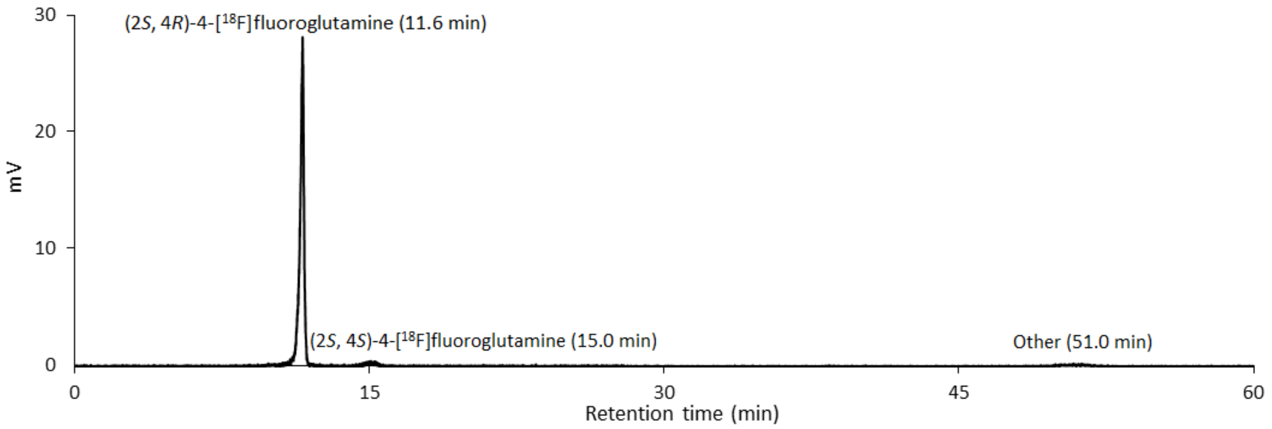

Supplemental Fig. 1. Sample quality control HPLC flow scintillation detector chromatogram of synthesized product containing 93.8% (2S, 4*R*)-4-[^{18}F]fluoroglutamine, 1.4% (2S, 4S)-4-[^{18}F]fluoroglutamine, and 4.8% other.

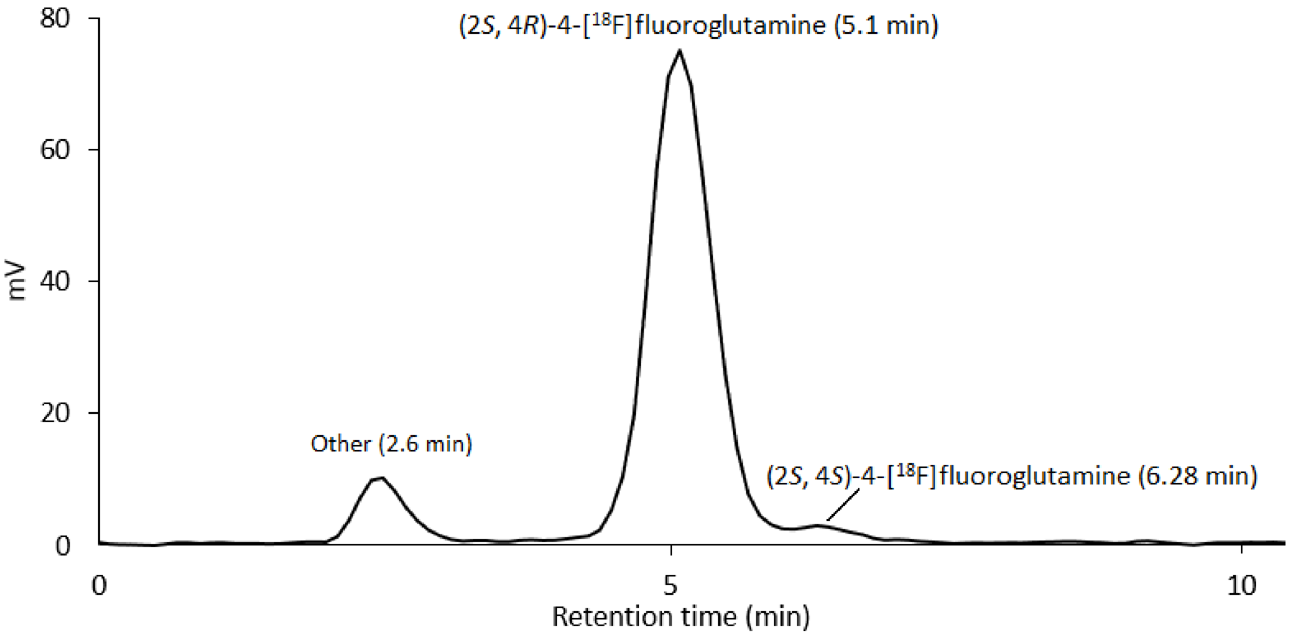

Supplemental Fig. 2. Sample stability analysis HPLC flow scintillation detector chromatogram.

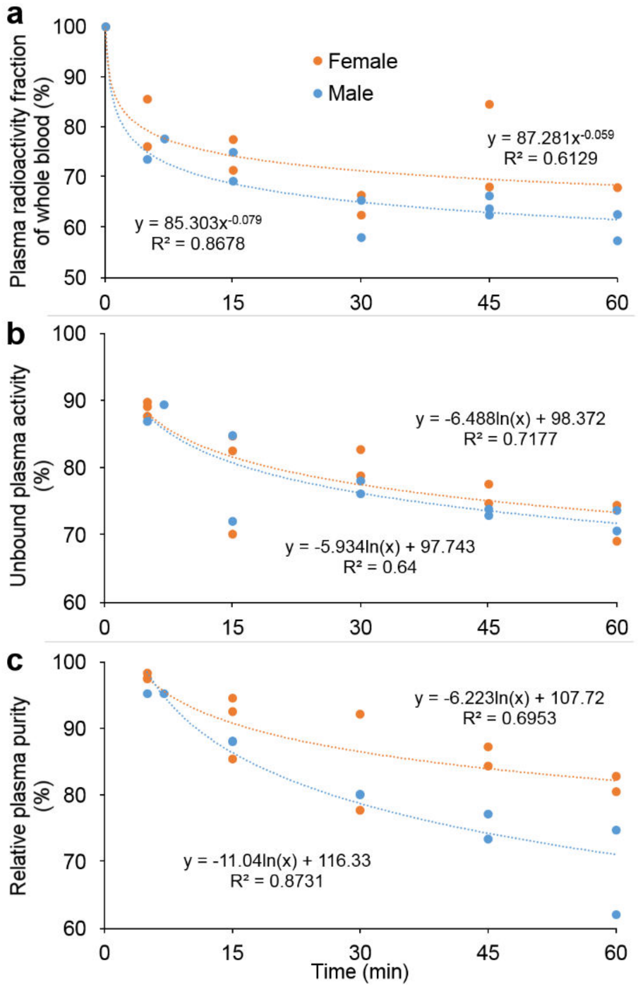

Supplemental Fig. 3. **a** Plasma fraction of the radioactivity (100-erythrocyte uptake %), **b** Unbound radioactivity % from precipitated plasma samples, and **c** Relative purity (adjusted to injection batch-purity as 100%) of the parent [^{18}F]FGln tracer in precipitated plasma supernatants.

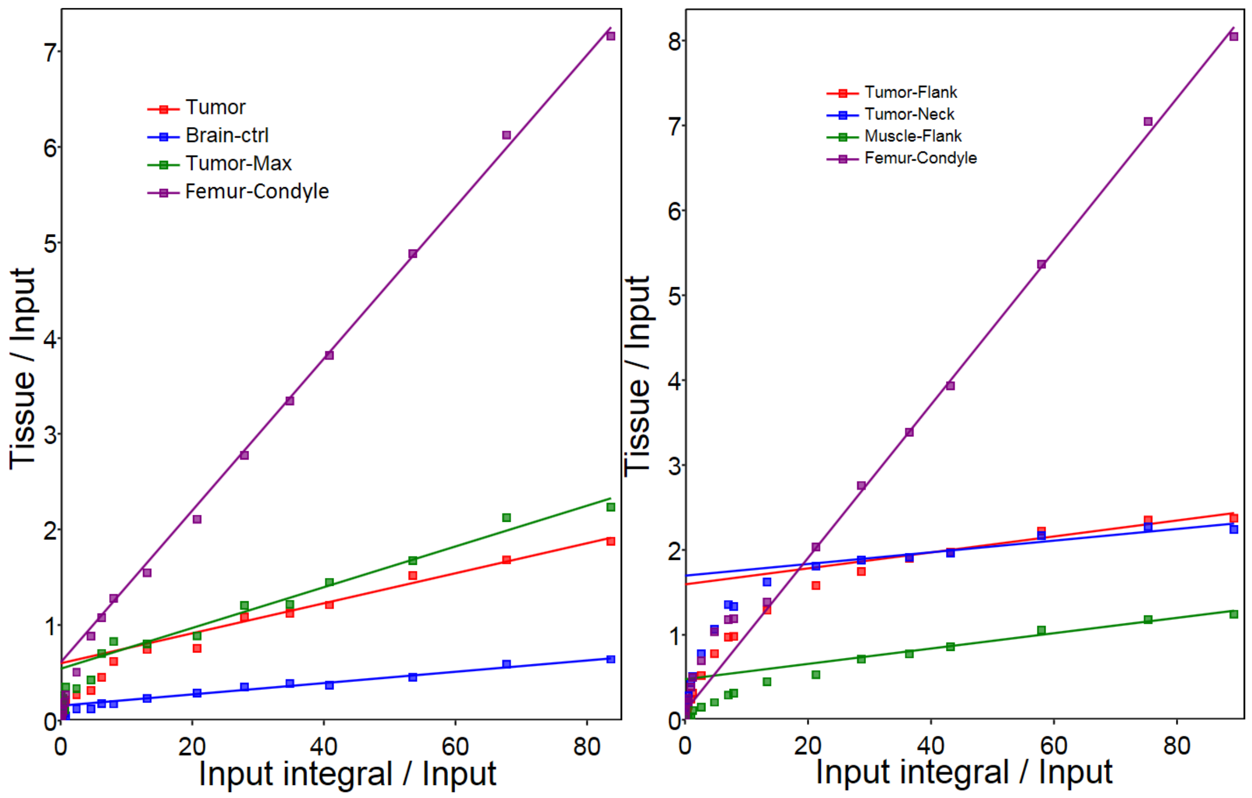

Supplemental Fig. 4. Patlak plot sample graphs. Left: arterial blood used as input, right: population based metabolite corrected fraction of arterial blood used as input.

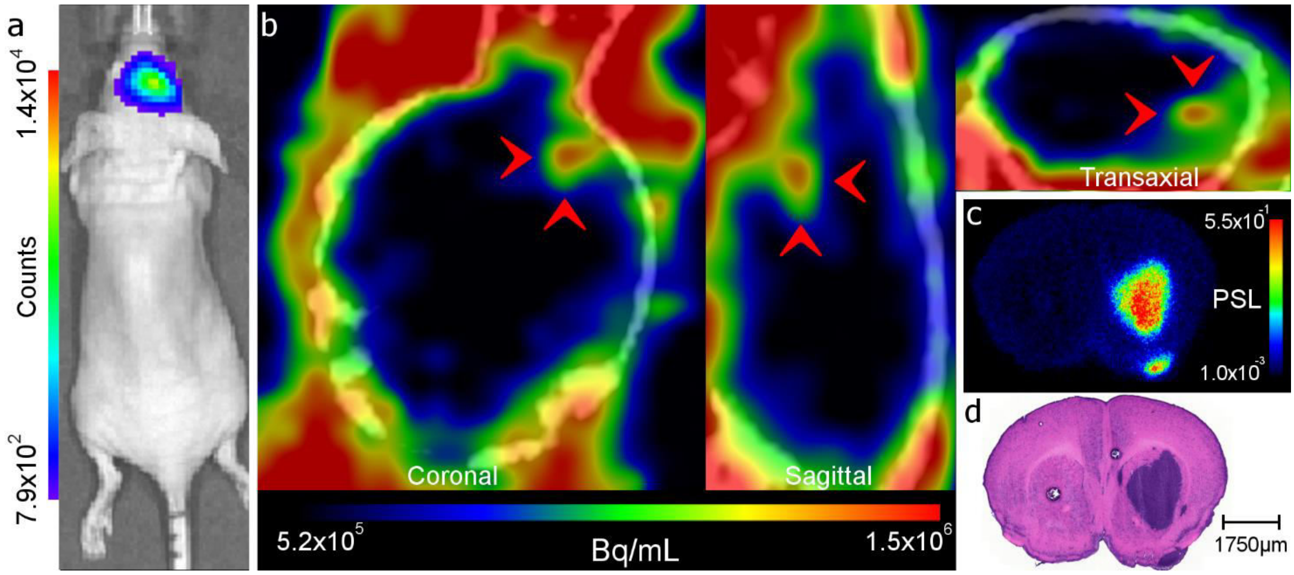

Supplemental Fig. 5. Multimodal image array of a BALB/cOlaHsd-Foxn1^{nu} mouse with a patient-derived BT3 glioma labeled with luciferase orthotopically grafted into the right hemisphere of the brain. **a** *In vivo* bioluminescence, **b** *In vivo* [^{18}F]FGln injected PET image slices (sum of frames from 5-25 min) with tumor indicated by red arrows, **c** *Ex vivo* autoradiograph of a (transaxial) cryosection, **d** light micrograph (hematoxylin-eosin stain) of the cryosection.

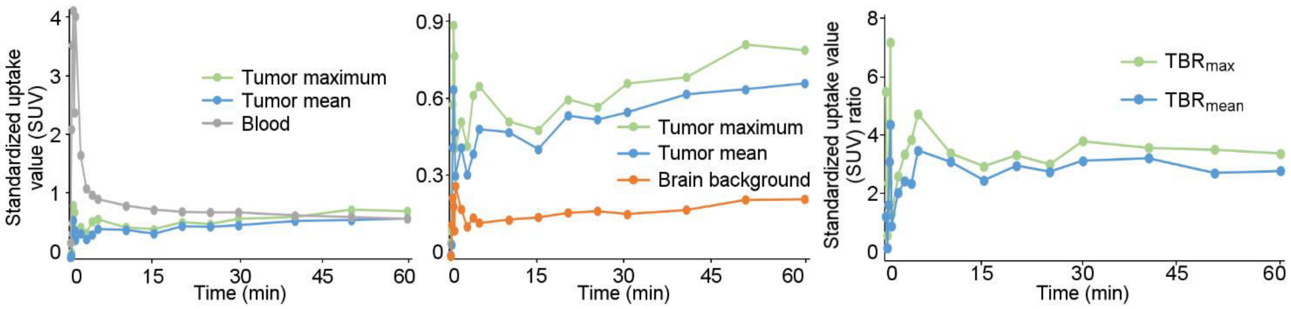

Supplemental Fig. 6. Array of PET time-activity curves for the [^{18}F]FGln injected BALB/cOlaHsd-Foxn1^{nu} mouse (PET images depicted in Supplemental Fig. 5b). TBR_{mean} = mean tumor radioactivity to mean brain background radioactivity ratio and TBR_{max} = maximum voxel radioactivity to mean brain background radioactivity ratio.

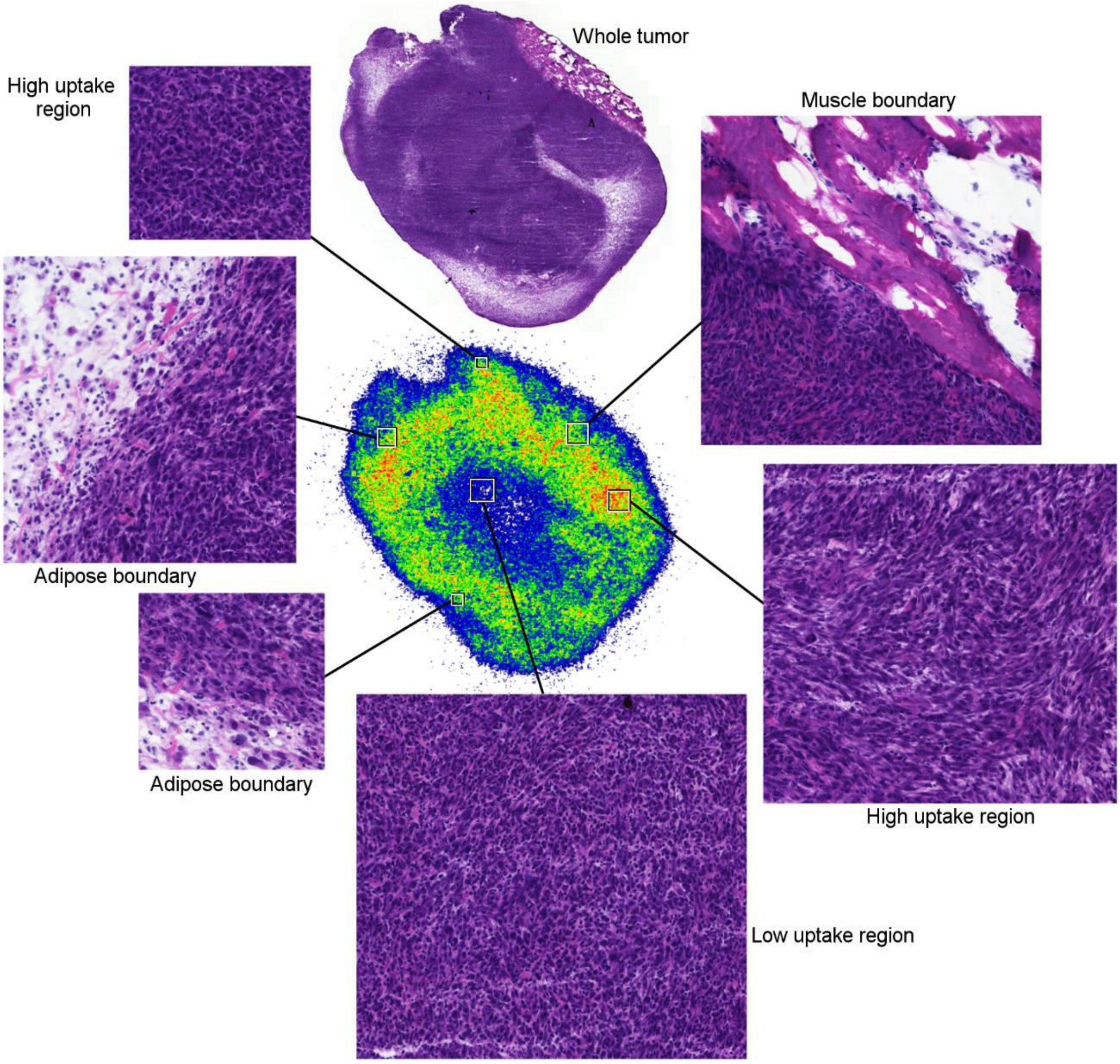

Supplemental Fig. 7. Central: *Ex vivo* autoradiograph of a subcutaneously grafted BT4C glioma exhibiting heterogenic [^{18}F]FGln uptake. Surrounding: Hematoxylin-eosin stain light micrographs of the whole 20 µm tumor slide and zoomed in areas indicated on the autoradiograph.

Supplemental Table 2. Patlak plot (irreversible uptake model) result summary for arterial blood and plasma-corrected fraction of arterial blood inputs

Parameter	Ki (AB)	Ic (AB)	r (AB)	Ki (PC)	Ic (PC)	r (PC)
Flank tumor average	0.0020	1.0521	0.4266	0.0082	1.5318	0.9629
(*n* = 6)	± 0.0034	± 0.2524	± 0.7869	± 0.0038	± 0.3888	± 0.0818
Flank muscle average	0.0033	0.5790	0.8361	0.0082	0.8154	0.9958
(*n* = 6)	± 0.0023	± 0.1835	± 0.3822	± 0.0028	± 0.2742	± 0.0091
Neck tumor average	0.0005	1.2083	0.3645	0.0069	1.7708	0.9443
(*n* = 6)	± 0.0025	± 0.1715	± 0.7187	± 0.0028	± 0.2668	± 0.1173
Bone (femoral condyle) average	0.0397	0.5752	0.9999	0.0688	0.3705	1.0000
(*n* = 6)	± 0.0119	± 0.1597	± 0.0001	± 0.0199	± 0.2561	± 0
Subject A o.t. glioma	0.0127	0.7386	0.9999	0.0257	0.9480	1.0000
Subject A glioma max.	0.010334	1.07	0.999	0.023587	1.4536	0.9999
Subject A brain (control)	0.0030	0.1654	0.9999	0.0060	0.2100	1.0000
Subject B o.t. glioma	0.0081	0.4687	0.9993	0.0157	0.5996	0.9998
Subject B glioma max.	0.011571	0.4556	0.9983	0.02131	0.5434	0.9993
Subject B brain (control)	0.0032	0.1316	0.9976	0.0059	0.1563	0.9992

AB = arterial blood activity used as input; PC = plasma corrected (population-based metabolite-corrected fraction of the arterial blood activity) used as input; Ki = net influx rate (min^{-1}), Ic = y intercept.

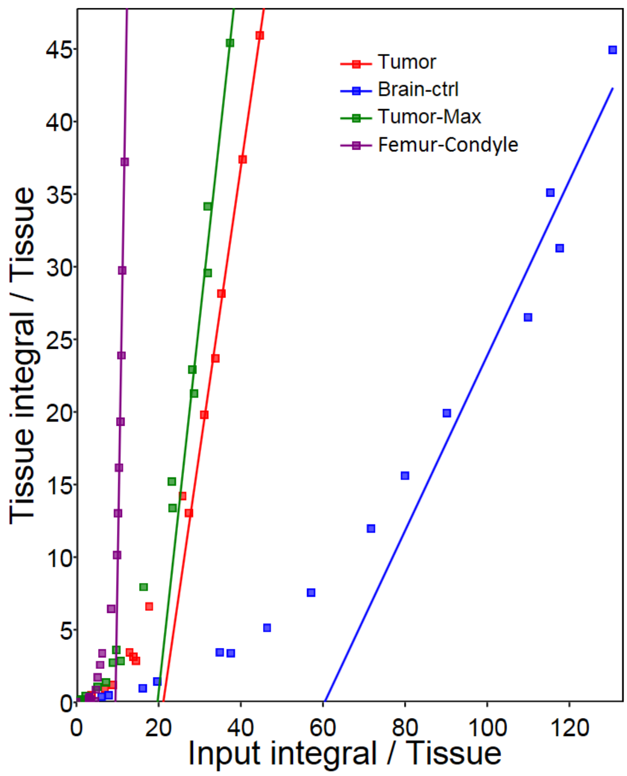

Supplemental Fig. 8. Logan plot sample graph with population-based metabolite-corrected fraction of arterial blood used as input.

Supplemental Table 3. Logan plot (reversible uptake model) result summary for arterial blood and plasma-corrected fraction of arterial blood inputs

Parameter	DV (AB)	Ic (AB)	r (AB)	DV (PC)	IC (PC)	r (PC)
Flank tumor average	1.13	-10.31	1.00	1.96	-19.79	1.00
(*n* = 6)	± 0.09	± 3.60	± 0	± 0.15	± 4.89	± 0
Flank muscle average	0.77	-15.28	1.00	1.38	-27.58	1.00
(*n* = 6)	± 0.13	± 5.34	± 0	± 0.23	± 7.76	± 0
Neck tumor average	1.21	-7.63	1.00	2.08	-16.47	1.00
(*n* = 6)	± 0.08	± 2.79	± 0	± 0.13	± 3.85	± 0
Bone (femoral condyle) average	5.11	-66.36	1.00	13.47	-142.79	0.86
(*n* = 6)	± 2.19	± 26.46	± 0.01	± 7.70	± 84.46	± 0.18
Subject A o.t. glioma	1.51	-19.69	1.00	2.97	-36.03	1.00
Subject A glioma max.	0.34	-18.72	1.00	0.67	-34.83	1.00
Subject A brain (control)	1.61	-14.15	1.00	2.97	-25.16	1.00
Subject B o.t. glioma	0.32	-20.23	1.00	1.95	-41.27	1.00
Subject B glioma max.	1.27	-26.85	1.00	0.60	-36.40	1.00
Subject B brain (control)	5.55	-61.91	1.00	2.57	-50.78	1.00

AB = arterial blood activity used as input; PC = plasma corrected (population-based metabolite-corrected fraction of the arterial blood activity) used as input; DV = distribution volume); IC = y intercept.

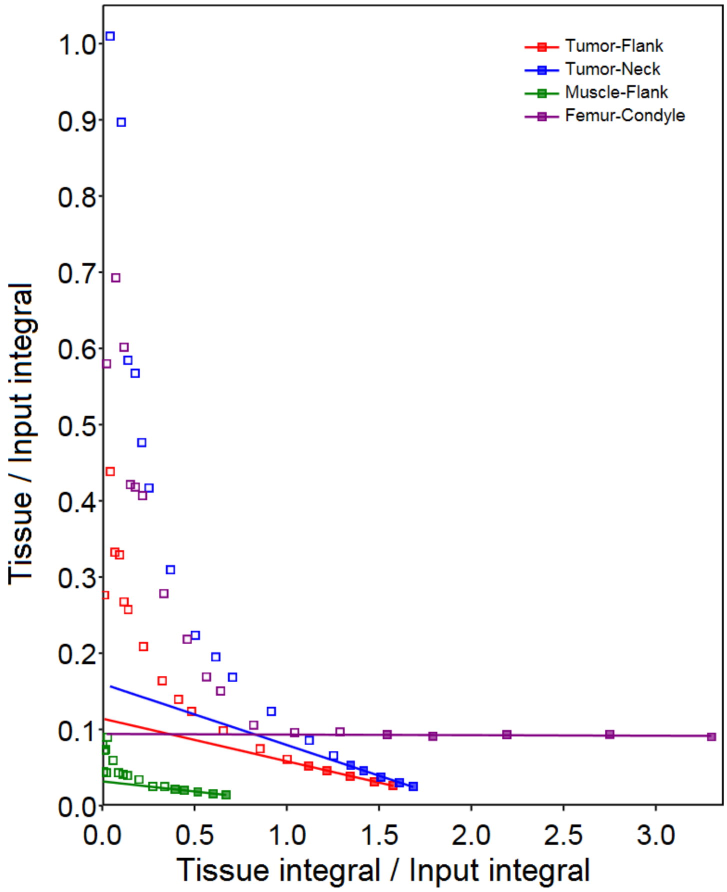

Supplemental Fig. 9. Sample Yokoi plot with population based metabolite corrected fraction of arterial blood used as input.

Supplemental Table 4. Yokoi plot result summary for arterial blood and plasma-corrected fraction of arterial blood inputs

Parameter	K1 (AB)	k2 (AB)	K1/k2 (AB)	K1 (PC)	k2 (PC)	K1/k2 (PC)
Flank tumor average (*n* = 6)	0.121 ± 0.039	0.106 ± 0.029	1.13 ± 0.09	0.105 ± 0.027	0.054 ± 0.011	1.95 ± 0.15
Flank muscle average (*n* = 6)	0.057 ± 0.023	0.074 ± 0.028	0.76 ± 0.13	0.054 ± 0.017	0.039 ± 0.013	1.37 ± 0.23
Neck tumor average (*n* = 6)	0.179 ± 0.076	0.146 ± 0.056	1.21 ± 0.08	0.135 ± 0.039	0.065 ± 0.016	2.08 ± 0.13
Bone (femoral condyle) average (*n* = 6)	0.075 ± 0.013	0.017 ± 0.012	5.48 ± 2.21	0.086 ± 0.015	0.005 ± 0.006	37.68 ± 39.62
Subject A o.t. glioma	0.078	0.052	1.50	0.083	0.028	2.96
Subject A glioma max.	0.120	0.075	1.59	0.121	0.041	2.95
Subject A brain (control)	0.018	0.054	0.34	0.019	0.028	0.68
Subject B o.t. glioma	0.045	0.046	0.99	0.047	0.024	1.96
Subject B glioma max.	0.046	0.036	1.29	0.049	0.017	2.79
Subject B brain (control)	0.015	0.045	0.33	0.015	0.022	0.69

AB = arterial blood activity used as input; PC = plasma corrected (Population-based metabolite-corrected fraction of the arterial blood activity) used as input; K1 = plasma to tissue transport rate (mL min^{-1} mL^{-1}); k2 = tissue to plasma transport rate (min^{-1}); K1/k2 = binding potential (mL mL^{-1}).

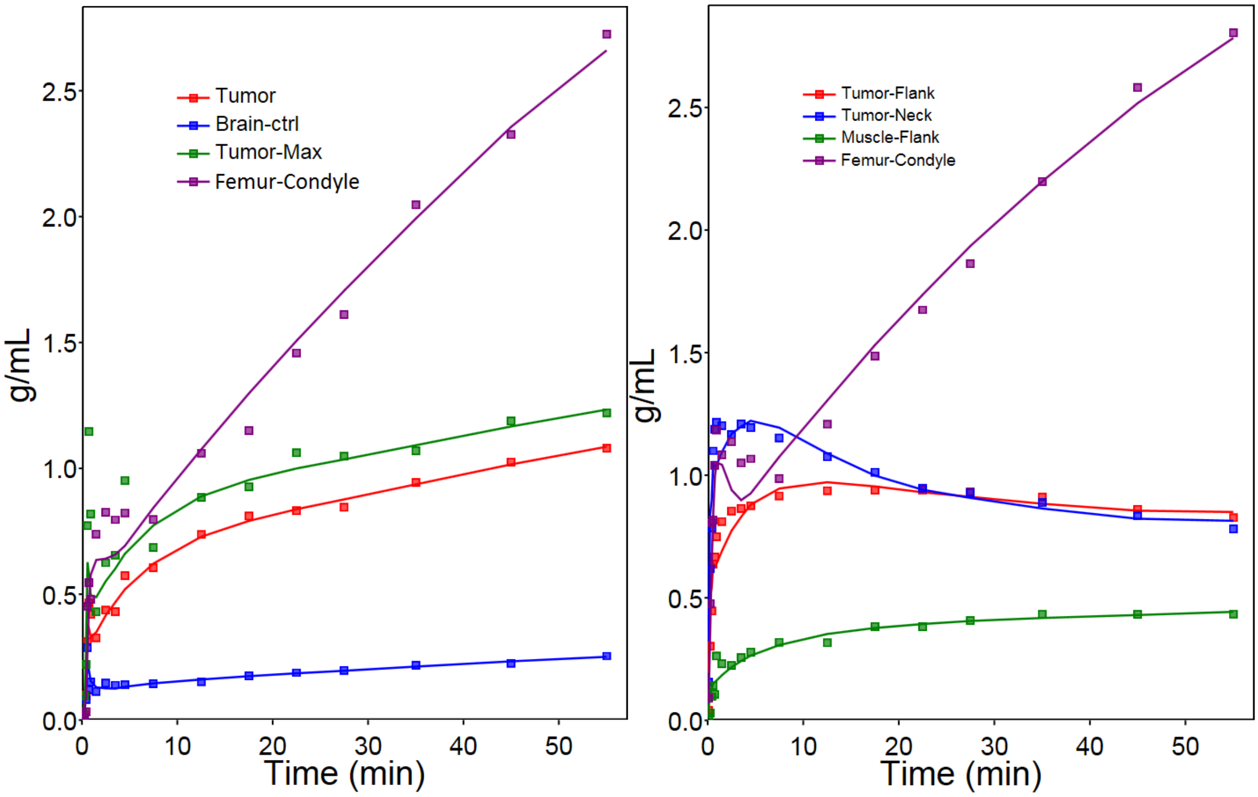

Supplemental Fig. 10. Sample 2-Compartment modeling plots of K1-k3 values from dynamic PET image tissue uptake data with arterial blood activity input from an orthotopic glioma bearing mouse (left) and mouse bearing subcutaneous tumors (right).

Supplemental Table 5. 2-Compartment model result summary for arterial blood as the input

Parameter	K1	K1/k2	k3	Vb	Ki	k3*K1/k2	k3/(k2+k3)	AIC
Flank tumor average	0.147	0.973	0.005	5.593	0.004	0.004	0.028	-122.617
(*n* = 6)	± 0.028	± 0.187	± 0.004	± 2.781	± 0.002	± 0.003	± 0.021	± 7.732
Flank muscle average	0.079	0.564	0.009	2.331	0.004	0.005	0.061	-131.200
(*n* = 6)	± 0.027	± 0.130	± 0.005	± 1.068	± 0.002	± 0.002	± 0.038	± 4.203
Neck tumor average	0.202	1.129	0.002	8.284	0.002	0.002	0.015	-120.760
(*n* = 6)	± 0.079	± 0.141	± 0.002	± 5.946	± 0.002	± 0.002	± 0.013	± 9.063
Bone (femoral condyle)	0.309	0.745	0.075	5.449	0.043	0.052	0.171	-99.013
average (*n* = 6)	± 0.179	± 0.194	± 0.034	± 2.532	± 0.014	± 0.017	± 0.083	± 9.576
Subject A o.t. glioma	0.116	0.919	0.018	11.514	0.014	0.016	0.122	-132.800
Subject A glioma max.	0.149	1.169	0.015	20.000	0.016	0.018	0.105	-87.870
Subject A brain (control)	0.015	0.143	0.032	7.822	0.003	0.005	0.235	-163.000
Subject B o.t. glioma	0.061	0.536	0.020	10.219	0.009	0.011	0.150	-111.100
Subject B glioma max.	0.102	0.447	0.036	13.278	0.014	0.016	0.137	-99.060
Subject B brain (control)	0.017	0.127	0.031	5.497	0.003	0.004	0.186	-154.800

K1 = plasma to tissue transport rate (mL min^{-1} mL^{-1}); k2 = tissue to plasma transport rate (min^{-1}); K1/k2 = binding potential (mL mL^{-1}), k3 = tissue to tissue transport rate (min^{-1}), Vb = distribution volume (mL), Ki = net influx rate (min^{-1}), AIC = Akaike Information Criteria

Supplemental Table 6. 2-Compartment model result summary for plasma-corrected fraction of arterial blood as the input

Parameter	K1	K1/k2	k3	Vb	Ki	k3*K1/k2	k3/(k2+k3)	AIC
Flank tumor average (*n*=6)	0.148 ± 0.027	1.369 ± 0.273	0.010 ± 0.004	6.636 ± 3.095	0.012 ± 0.002	0.013 ± 0.003	0.086 ± 0.028	-116.683 ± 8.077
Flank muscle average (*n*=6)	0.081 ± 0.027	0.797 ± 0.163	0.015 ± 0.005	2.796 ± 1.117	0.010 ± 0.003	0.012 ± 0.003	0.136 ± 0.055	-128.083 ± 6.250
Neck tumor average (*n*=6)	0.202 ± 0.072	1.567 ± 0.209	0.008 ± 0.002	9.786 ± 6.848	0.012 ± 0.001	0.013 ± 0.002	0.065 ± 0.026	-113.280 ± 8.946
Bone (femoral condyle) average (*n*=6)	0.402 ± 0.206	0.649 ± 0.191	0.148 ± 0.063	3.213 ± 3.573	0.069 ± 0.019	0.086 ± 0.024	0.198 ± 0.079	-98.883 ± 14.434
Subject A o.t. glioma	0.123	1.373	0.027	11.636	0.029	0.038	0.234	-132.700
Subject A glioma max.	0.156	1.769	0.023	20.000	0.032	0.041	0.208	-87.840
Subject A brain (control)	0.016	0.210	0.051	7.770	0.006	0.011	0.396	-163.100
Subject B o.t. glioma	0.063	0.767	0.032	10.489	0.018	0.025	0.280	-110.900
Subject B glioma max.	0.111	0.515	0.062	13.358	0.025	0.032	0.224	-99.440
Subject B brain (control)	0.018	0.168	0.051	5.519	0.006	0.009	0.318	-154.900

K1 = plasma to tissue transport rate (mL min^{-1} mL^{-1}); k2 = tissue to plasma transport rate (min^{-1}); K1/k2 = binding potential (mL mL^{-1}), k3 = tissue to tissue transport rate (min^{-1}), Vb = distribution volume (mL), Ki = net influx rate (min^{-1}), AIC = Akaike Information Criteria

Ex Vivo Biodistribution Results

The tracer accumulated highly in the pancreatic tissue with notable high uptake in the skull (bone) and femur (bone + marrow). The bone uptake is classically characteristic of defluorination occurring which leads to absorption into the osseous tissue. Uptake in brown adipose tissue is significantly higher than white adipose tissue likely due to the comparative increased number of metabolic processes which encompass its cell type characteristics.

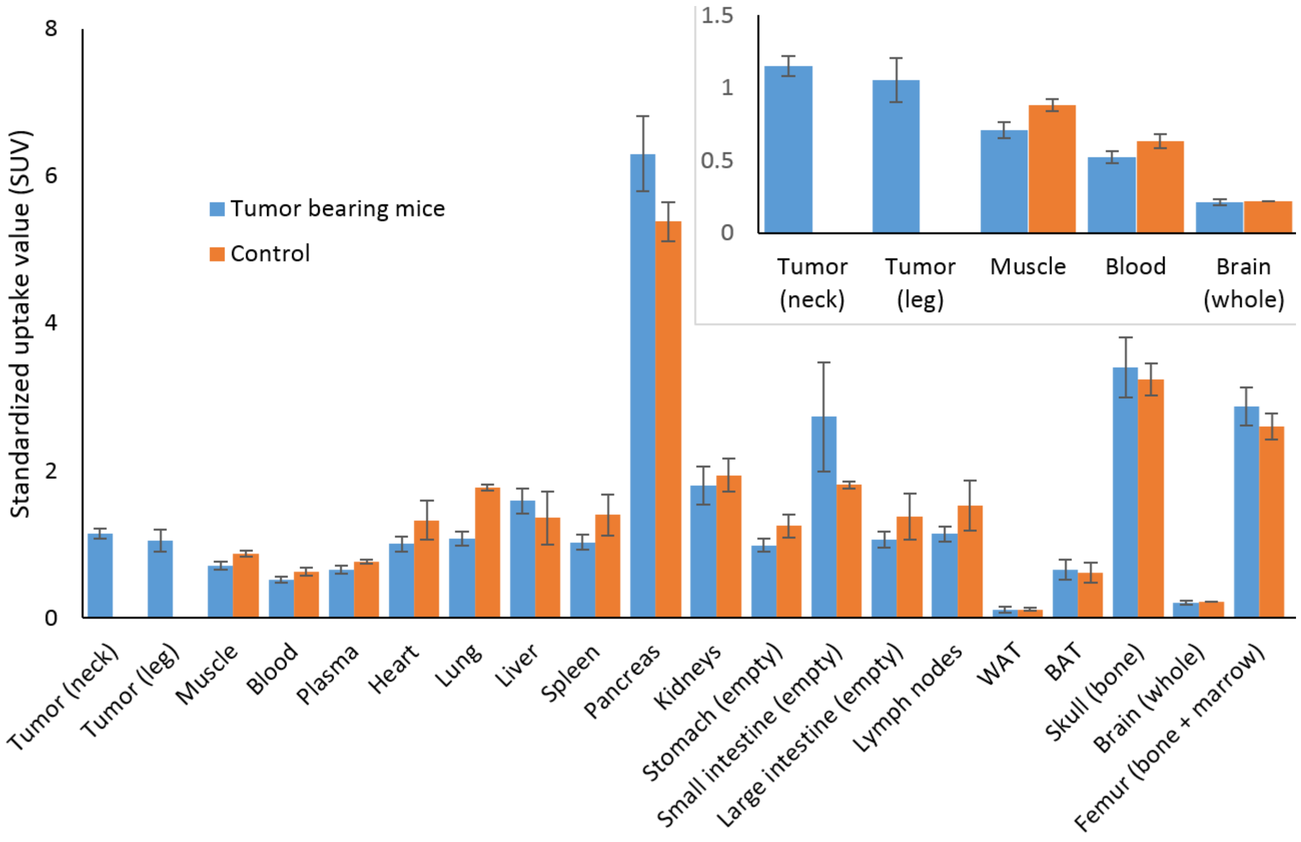

Supplemental Fig. 11. *Ex vivo* biodistribution averages of [^{18}F]FGln uptake 63 minutes post-injection in subcutaneously xenografted BT4C tumor bearing Foxn1$^{nu/nu}$ mice (female, *n* = 5) and control C57BL/6JRj mice (male, *n* = 2).
